# Association between Hippocampal Volume and Cognition in the Oldest‐Old Individuals from Colombia

**DOI:** 10.1002/alz.090936

**Published:** 2025-01-03

**Authors:** Elizabeth Kaplan, Yesica Zuluaga, Daniel Vasquez, Liliana Hincapie, David Fernando Aguillon, Lucia Madrigal, Ana Y Baena, Clara Vila‐Castelar, Sergio Alvarez, Martin Ochoa‐Escudero, Margarita M. Giraldo‐Chica, Liliana A Ramirez Gomez, Stephanie Langella, Joseph F Arboleda‐Velasquez, Francisco Lopera, Yakeel T. Quiroz

**Affiliations:** ^1^ Massachusetts General Hospital, Harvard Medical School, Boston, MA USA; ^2^ Grupo de Neurociencias de Antioquia, Universidad de Antioquia, Medellín Colombia; ^3^ Grupo de Neurociencias de Antioquia, Facultad de Medicina, Universidad de Antioquia, Medellín, Antioquia Colombia; ^4^ Hospital Pablo Tobón Uribe, Medellín Colombia; ^5^ Schepens Eye Research Institute of Mass Eye and Ear and Department of Ophthalmology, Harvard Medical School, Boston, MA USA; ^6^ Grupo de Neurociencias de Antioquia, Facultad de Medicina, Universidad de Antioquia, Medellín Colombia

## Abstract

**Background:**

Life expectancy is on the rise, accompanied by an increased prevalence of cognitive impairment and Alzheimer’s disease. Despite this global trend, our understanding of the neural mechanisms underlying cognitive changes in the oldest‐old (>80 years old) population remains limited. Unraveling these mechanisms may provide valuable insights and therapeutic interventions for individuals grappling with cognitive impairments in older age. Increased hippocampal volume has been associated with better cognitive function and its sparing in late life has been linked to better cognitive and functional outcomes. In this study, we examined hippocampal volume and cognitive performance in a group of the oldest‐old individuals from Colombia.

**Methods:**

13 participants (9 women, 4 men), with an average age of 94.8 (6.24) years were included in this study. Cognitive performance was measured using the Mini‐Mental State Examination (MMSE) and the Consortium to Establish a Registry for Alzheimer’s Disease (CERAD) Word List Learning Total. Functional impairment was assessed using the Functional Assessment Scale Test (FAST). Structural MRI scans were conducted at the Hospital Pablo Tobon Uribe in Colombia and processed with FreeSurfer (v7.2). Right and left hippocampal volumes were averaged. Linear regression models were used to investigate the relationship between hippocampal volume and cognitive performance.

**Results:**

7 participants were cognitively‐unimpaired (FAST = 1), 3 endorsed subjective complaints (FAST = 2), and 3 met criteria for dementia (FAST ≥3). Participants had a mean hippocampal volume of 2,990 (459 mm³), mean MMSE score of 21.2 (6.15), and mean Memory Score of 10.5 (4.52). Regression models revealed higher hippocampal volume was significantly associated with better memory scores after adjusting for FAST impairment, age, sex, and intracranial volume (p = 0.012). The relationship between higher hippocampal volume and MMSE scores, controlling for the same variables, approached significance (p = 0.06).

**Conclusions:**

Our preliminary findings suggest a positive association between hippocampal volume, a marker of neurodegeneration, and cognitive performance in a cohort of the oldest‐old individuals from Colombia. This study is ongoing and future research, incorporating a larger sample size and additional biomarkers, is needed to validate these results, as well as to further our understanding of the relationship between neural structure in late life and cognition.

